# Willingness to Self-Collect a Sample for HPV-Based Cervical Cancer Screening in a Well-Screened Cohort: HPV FOCAL Survey Results

**DOI:** 10.3390/curroncol29060308

**Published:** 2022-05-26

**Authors:** Anne Lesack, Laurie W. Smith, C. Sarai Racey, Lovedeep Gondara, Mel Krajden, Marette Lee, Ruth Elwood Martin, Gavin Stuart, Stuart Peacock, Eduardo L. Franco, Dirk van Niekerk, Gina S. Ogilvie

**Affiliations:** 1 Faculty of Medicine, University of British Columbia, Vancouver, BC V6T 1Z4, Canada; anne.lesack@cw.bc.ca (A.L.); sarai.racey@cw.bc.ca (C.S.R.); mel.krajden@bccdc.ca (M.K.); marette.lee@vch.ca (M.L.); ruth.martin@ubc.ca (R.E.M.); gavin.stuart@ubc.ca (G.S.); dvanniek@bccancer.bc.ca (D.v.N.); gina.ogilvie@bccdc.ca (G.S.O.); 2 Cancer Control Research, BC Cancer, Vancouver, BC V5Z 1L3, Canada; speacock@bccrc.ca; 3 Women’s Health Research Institute, BC Women’s Hospital, Vancouver, BC V6H 3N1, Canada; 4 Cancer Surveillance and Outcomes, BC Cancer, Vancouver, BC V5Z 4E6, Canada; lovedeep.gondara@bccancer.bc.ca; 5 Public Health Laboratory, BC Centre for Disease Control, Vancouver, BC V5Z 4R4, Canada; 6 Cervix Screening Program, BC Cancer, Vancouver, BC V5Z 4E6, Canada; 7 Faculty of Health Sciences, Simon Fraser University, Vancouver, BC V5A 1S6, Canada; 8 Division of Cancer Epidemiology, Faculty of Medicine, McGill University, Montreal, QC H4A 3T2, Canada; eduardo.franco@mcgill.ca

**Keywords:** human papillomavirus, HPV, HPV self-sampling, HPV testing for cervix screening, attitudes and acceptance HPV self-sampling

## Abstract

Self-collection may provide an opportunity for innovation within population-based human papillomavirus (HPV) cervical cancer screening programs by providing an alternative form of engagement for all individuals. The primary objective was to determine willingness to self-collect a vaginal sample for primary HPV screening and factors that impact willingness in individuals who participated in the Human Papillomavirus For Cervical Cancer (HPV FOCAL) screening trial, a large randomized controlled cervical screening trial. A cross-sectional online survey was distributed between 2017 and 2018 to 13,176 eligible participants exiting the FOCAL trial. Bivariate and multivariable logistic regression assessed factors that influence willingness to self-collect on 4945 respondents. Overall, 52.1% of respondents indicated willingness to self-collect an HPV sample. In multivariable analysis, the odds of willingness to self-collect were significantly higher in participants who agreed that screening with an HPV test instead of a Pap test was acceptable to them (odds ratio (OR): 1.45 (95% confidence interval (CI): 1.15, 1.82), those who indicated that collecting their own HPV sample was acceptable to them (*p* < 0.001), and those with higher educational ascertainment (OR: 1.31, 95% CI: 1.12, 1.54). The findings offer insight into the intentions to self-collect in those already engaged in screening, and can inform cervical cancer screening programs interested in offering alternative approaches to HPV-based screening.

## 1. Introduction

Human papillomavirus (HPV) is one of the most common sexually transmitted infections (STI) globally [1]. Persistent infection with a high-risk oncogenic HPV (hr-HPV) genotype has been well established as a necessary cause for most cervical cancer cases [2]. Although prophylactic HPV vaccines are widely available and a key component of cervical cancer prevention [3], screening for cervical cancer will remain an integral form of prevention for the foreseeable future [4].

Historically, cytology testing has been, and continues to be, the standard of care for cervical cancer screening in many jurisdictions with organized screening programs [5]. Currently in British Columbia (BC), cytology (conventional Pap) testing is recommended every 3 years for those between 25–69 years of age [6]. However, cervical cancer screening is undergoing a paradigm shift towards primary HPV testing, due to its increased sensitivity and effectiveness for detecting cancerous and pre-cancerous lesions—cervical intraepithelial neoplasia (CIN) grade 2 or worse (CIN2+) [7,8].

A major consideration for primary HPV screening is that the exfoliated cellular sample does not need to be collected directly from the cervix, enabling the sample to be self-collected vaginally. Screening performance, i.e., sensitivity and specificity, is comparable between self-collected and practitioner-collected samples for HPV screening [9]. Therefore, self-collection has the potential to improve coverage and convenience in HPV-based screening programs [9,10]. Screening programs in Australia [11], the Netherlands [12], and Sweden [13] have begun adopting self-collection, predominantly offering self-collection to people who do not routinely participate in screening and are therefore at higher risk for cervical cancer. The Netherlands also offers self-collection to those who do not feel comfortable receiving screening from a provider and would otherwise not participate in screening [12].

Self-collection provides an avenue for increased accessibility to cervical cancer screening and is seen to improve screening rates for individuals who are typically under-screened by reducing logistical and personal barriers encountered with clinician-based screening [14,15,16,17]. Furthermore, self-collection has the potential to offer a more convenient and acceptable form of screening for all individuals, including those who are already engaged in routine screening.

Populations that adhere to screening are often fundamentally different from those who do not. Findings from acceptability studies on under-screened individuals may not therefore be generalizable to the well-screened population. There is currently a paucity of data regarding the acceptability of self-collection in the well-screened population. For the successful integration of self-collection in screening programs for all screen-eligible individuals, there is a need to evaluate willingness and acceptability of self-collection in the well-screened population.

The primary objective of this analysis was to determine willingness to self-collect a vaginal sample for primary HPV screening in future among participants of the BC Human Papillomavirus For Cervical Cancer (HPV FOCAL) screening trial. Factors associated with a preference for self-collection were also measured in this well-screened cohort.

## 2. Materials and Methods

HPV FOCAL was a three-armed, randomized control trial, with the primary goal of establishing the effectiveness of HPV-based screening compared to the standard of care, cytology (liquid-based), in the detection of CIN2+ within an organized screening program [7,18,19,20]. Participants in the BC Cervix Screening Program who were due for screening and were patients of collaborating clinicians in Metro Vancouver and Greater Victoria were invited to participate [7]. Recruitment occurred from 2008 to 2012. A total of 19,009 participants between the ages of 25 to 65 were recruited into the control (cytology) and intervention (HPV) arms of the trial. Throughout the trial, participants received information regarding the natural history of HPV, the difference between HPV and cytology (Pap) testing, and the meaning of test results.

HPV FOCAL participants in the control or intervention arms completed the trial with exit screening between 2012 and 2016, where they received co-testing with clinician-collected cytology and HPV testing. Between August 2017 and February 2018 an online exit survey (File S1) was distributed to participants who completed the 48-month exit screen [21]. A total of 14,535 individuals were invited to complete the exit survey.

### 2.1. Exit Survey

The exit survey contained demographic questions, and other questions regarding perceptions of Pap versus HPV testing, sources of knowledge, and hypothetical questions regarding the acceptability of HPV-based self-collection. Survey questions were based on previous surveys administered throughout the HPV FOCAL trial and knowledge from the literature around known risk factors. Responses were recorded on either 5-point or 7-point Likert scales [21]. Participants were asked about their willingness to self-collect a vaginal sample after being provided with a short description of self-collection. Participants were not provided any visual aids depicting the process of self-collection, nor did they have the opportunity to undergo self-collection themselves.

The survey was distributed and responses collected through the online survey platform FluidSurveys. The survey was pilot tested on 20 women, aged 30 years and above, to assess both face and content validity before distribution to the study population.

### 2.2. Response Rate and Inclusion Criteria

All participants in both the cytology and HPV arms who had completed a 48-month exit screening and provided consent to be contacted for future research with an email address were eligible to receive the exit survey. Participants were sent a survey link via email and with a reminder at 1 month if they had not initiated or completed the survey [21].

Upon survey closure, duplicate and incomplete responses were checked. For participants who submitted duplicate responses, the first completed survey was kept, and the additional responses were discarded. Response rate was calculated using the American Association for Public Opinion Guidelines, dividing the total number of complete and partial surveys by the total number of surveys distributed to valid email addresses [22].

### 2.3. Statistical Model

The primary outcome of interest was response to the statement “I would be willing to collect my own sample/specimen for cervical cancer screening”. Responses to this statement were recorded on a 5-point Likert scale (strongly disagree to strongly agree). Responses were dichotomized to either “willing” or “not willing”; those who agreed and strongly agreed to the statement were classified as willing, as all those who were neutral, disagreed or strongly disagreed, or ‘did not know’, were categorized as not willing. Responses were categorized in such a way as to capture a conservative estimate of those who were truly willing to self-collect. Bivariate and multivariable analysis were based on complete case analysis, due to minimal missing data.

Demographic variables, variables associated with, or those that might impact, willingness to complete future self-collection were assessed through bivariate analysis. Variables were selected based on *a priori* knowledge and relationships identified in the literature. Chi-square and Fishers exact test (where applicable) were used to assess categorical relationships and the Wilcoxon rank-sum test was used for continuous variables.

Multivariable logistic regression was used to assess the influence of *a priori* selected variables on the outcome of willingness to complete self-collection. All variables in the bivariate analysis were included after confirmation of the absence of collinearity. The threshold of significance was a *p* value < 0.05. R software version 4.1.0 [23] was used to conduct all statistical analysis.

## 3. Results

**Figure 1:**
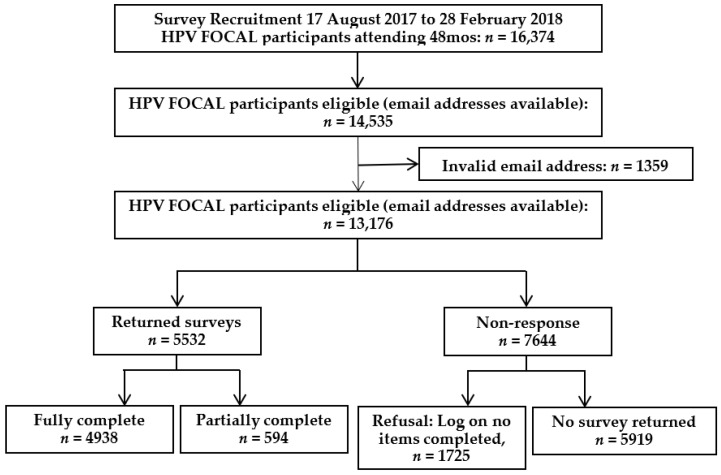
Study flow chart.

The HPV FOCAL exit survey was distributed between August 2017 and February 2018 to 14,535 participants who completed the trial exit screen with an email address, of whom 13,176 had a valid email address to which the survey did not bounce back. A total of 5532 (42%) participants returned a survey, 4945 of whom answered the questions regarding willingness to self-collect, and are included in this analysis (Figure 1).

### 3.1. Characteristics of Study Population

The median age of respondents was 53.8 (interquartile range (IQR): 45.4,61.5) (Table 1). Most participants had completed college or achieved a higher level of education (66.9%). Most participants (76.7%) lived with a partner, and over 60% had a household income over $75,000 CAD annually. The exit survey respondents were comparable to the HPV FOCAL trial participants based on geography and age [21].

### 3.2. Willingness to Self-Collect

Overall, 52.1% of respondents indicated that they would be willing to collect their own sample for HPV-based cervical cancer screening; 12.1% of study respondents provided a neutral response, making up 25.2% of those categorized as not willing to self-collect. Participants who were older, had completed a higher level of education, and those who made over $75,000 annually were significantly more willing to self-collect. There was no significant association between marital status and willingness to self-collect. Respondents who reported HPV testing for cervical cancer screening to be acceptable compared to Pap testing (62.8%), and those who reported self-collection to be acceptable (67.9%), were more willing to participate in self-collection in the future.

### 3.3. Multivariable Results

In multivariable analysis, participants who were older were more willing to self-collect a vaginal sample (OR: 1.01 (95% confidence interval (CI): 1.00, 1.02), *p* = 0.002); however, the effect size is likely negligible (Table 2). Respondents who completed college or a higher level of education were significantly more willing to self-collect compared to their incomplete post-secondary or lower counterparts, controlling for all other variables (*p* < 0.001). Marital status and household income were not found to be significant predictors of willingness to self-collect a sample. Respondents who answered neutrally to the statement “I know more about HPV and cervical cancer now than I did before I participated in the study”, were significantly less willing to self-collect compared to those who indicated they disagreed with the statement. Participants who agreed with the statement, indicating they knew more about HPV and cervical cancer at study close, were no more willing to self-collect than those who felt they did not know more about HPV after study participation. The odds of willingness to self-collect were higher in participants who agreed that screening with an HPV test instead of a Pap test was acceptable to them compared to those who were unaccepting of HPV-based screening (OR: 1.45 (95% CI: 1.15, 1.82), *p* = 0.002). Respondents who indicated that collecting their own HPV sample was acceptable to them were significantly more willing to self-collect compared to those who indicated that they found self-collection to be unacceptable (*p* < 0.001).

## 4. Discussion

Willingness to self-collect a sample for HPV-based cervical cancer screening was explored in a cohort of 4945 individuals who had undergone HPV and cytology testing as part of the HPV FOCAL trial, a randomized controlled trial embedded within an organized screening program. Just over half of participants (52.1%) indicated that they would be willing to undergo self-collection for HPV testing in the future. This is a conservative estimate of willingness to participate in self-collection in this cohort, given the classification of ‘willingness’ in our analysis was purposely biased towards the null by only including participants as ‘willing’ if they used the emphatic responses of ‘willing or very willing’. In a sensitivity analysis, ‘willingness to self-collect’ increased to 64.1% in this cohort if those with a neutral response were included in the definition of ‘willing’, as opposed to ‘unwilling’.

Participants who reported that self-collection was acceptable and were accepting of HPV-based screening compared to the Pap test for cervical cancer screening were more willing to complete self-collection in the future. Willingness to self-collect was higher in the exit survey respondents compared to a sample of FOCAL trial participants surveyed in 2011 [20].

In multivariable analysis, participants who were older and had a higher level of education were significantly more willing to self-collect a vaginal sample. There was no association between income and willingness to self-collect. In existing literature, there is limited evidence for strong and consistent associations between self-collection acceptability and key demographic characteristics, indicating a similar level of acceptance for self-collection across subsets of the population [16]. A systematic review identified no association between age across studies [16], although, in a meta-analysis conducted by Yeh et al., a slightly stronger uptake of self-collection was seen in women over 50 years of age [24]. Within the HPV FOCAL exit survey, respondents were older than the general screening population within BC [25]. The difference in median age between willing and unwilling groups was just under 1 year apart, and although this difference is statistically significant, it is also negligible and will likely not have any impact on screening programs and outcomes (Table 1). Within the wider BC population, it is likely that there is high acceptability across all age groups that has not been captured due to the characteristics of the respondent population.

Similar to age, the level of educational attainment has rarely been identified as a significant predictor of acceptance of self-collection in the literature [16]. Two studies identified a significant association, but lack comparability to the HPV FOCAL population due to the rural context and overall low education level of the populations [26,27]. As seen with age, a significant relationship may be observed in the HPV FOCAL cohort due to the disproportionately high level of educational attainment within this study population compared to the wider BC population.

Most of the published literature exploring acceptance of self-collection has rightly focused on individuals who do not receive regular screening or have never received screening, and for whom structural, personal, and logistical barriers hinder access to screening. However, there is a paucity of data regarding acceptability or willingness to use self-collection as an approach to HPV-based screening for those who routinely engage in cervical cancer screening.

In BC, around 70% of women undergo screening according to guidelines [25]. As HPV testing is introduced into cervical cancer screening guidelines and there is the potential to adopt self-collection, there is a strong need for data on the acceptability of self-collection in the majority of women who currently access screening. Data on the acceptability of self-collection among well screened women will be relevant to other jurisdictions with existing screening programs [28]. The HPV FOCAL exit survey respondents comprise a sample of individuals who regularly participate in routine cervical cancer screening provided by their health care provider and are able to provide an important perspective on the acceptability of vaginal self-collection within the BC context [20].

In this study, over 60% of participants agreed that having an HPV test to screen for cervical cancer instead of a Pap smear was acceptable to them and over 65% of participants indicated that collecting their own sample for cervical cancer screening was acceptable to them. The high acceptability of self-collection indicated by the FOCAL exit survey respondents aligns with high acceptance demonstrated by under-screened populations [15,17,29,30]. In under-screened groups, the ability of self-collection to overcome personal and practical barriers contributes to its high acceptability; it is seen as less embarrassing, more private, less painful, and takes less time overall than clinician-collected sampling [19,20,21]. High acceptance of self-collection among the HPV FOCAL cohort may be seen in part due to these logistical and personal benefits, offering a more convenient and comfortable form of screening for all individuals. High acceptability of self-collected vaginal swabs for cervical cancer screening in both highly screened and under-screened populations may help improve overall engagement and coverage of cervical cancer screening.

Self-collection provides an opportunity for innovation within current population-based screening programs. In addition to reducing personal and practical barriers to screening [15], self-collection has the unique opportunity to also address emerging barriers to screening such as in public health emergencies, or other situations that affect face-to-face services in the health system. Broadening organized screening programs through the integration of self-collection aligns with the WHO directives around self-care and autonomy in health care [31], in part due to its ability to overcome these barriers. Offering increased access to self-care empowers individuals and allows individuals to make more informed decisions around their health care, simultaneously increasing access and equity in health care attainment [31,32]. In light of the COVID-19 pandemic, a push to implement self-care driven models may occur due to widened exposure to these models in health care settings over the course of the pandemic. Self-collection decreases the demand on the health care system for face-to-face care and increases access to those who need screening; however, systems need to be in place to ensure that those who require follow-up care have access to timely clinical care.

There are numerous factors that ultimately contribute to the uptake of preventative health behaviors. Study findings indicate that individuals who agreed that they had more knowledge of HPV and cervical cancer at the end of the study were not more willing to self-collect compared to those who did not feel they had gained more knowledge over the trial period. The exit survey respondents were a highly educated cohort; it is possible that this may influence some respondents’ acceptability of alternative approaches to health care and screening, regardless of knowledge acquired during the study. It is possible that recall bias may have been introduced as exit surveys were distributed on average 3 years after participants completed the HPV FOCAL trial, potentially leading respondents to inaccurately recall knowledge acquisition during the trial [21]. Lastly, although knowledge is an important piece in the pathway to action, it is not the only factor that contributes to the uptake of a behavior; emotional and social factors as well as perceived risk and barriers all play an important role in determining uptake of a preventative behavior [33].

The HPV FOCAL exit survey results, in combination with results from previous studies of under-screened populations, indicate that HPV-based self-collection is acceptable by all individuals eligible for cervix screening, and that offering self-collection in combination with clinician collected sampling could increase screening rates within the BC context.

### Limitations

This study has some key limitations to consider. All participants of the HPV FOCAL trial who completed the 48-month exit screen were eligible for the FOCAL exit survey; however, not all trial participants had eligible email addresses and the overall exit survey response rate was 42%, indicating there was the potential for non-response bias. However, a comparison of respondents and non-respondents indicated that the populations were not significantly different [21].

The HPV FOCAL cohort has been well characterized [7,21], and compared to all individuals eligible for cervix screening in BC, respondents to the exit survey were highly educated, of high socioeconomic status, and lived in two major urban centers. Due to the homogeneity of the study population, these findings may lack generalizability to more economically and regionally diverse populations. However, study participants do reflect the current population receiving cytology testing through the provincial screening program; their perspectives can therefore inform programs interested in offering self-collection as an alternate form of screening.

Previous studies have predominantly captured reported acceptance of self-collection after participants were offered or completed self-collection; however, in our study we asked about future willingness to collect. It is possible that our measure of willingness and acceptability is either overestimated or underestimated due to participants not having the ability to experience the process first-hand. Lastly, prior to asking the questions regarding self-collection, a very brief description of vaginal self-collection was provided in the survey, with no information shared regarding devices or instructions. This lack of contextual information may have impacted how respondents reported their willingness to self-collect at the time of the survey. Despite these limitations, these study findings provide an important first step in building an understanding of willingness and acceptability of self-collection within a well-screen population.

## 5. Conclusions

These findings offer insight into willingness to self-collect in those already engaged in screening and can inform programs interested in offering alternative approaches to HPV-based screening. Further intention and implementation research is required to understand how best to introduce self-collection, and how self-collection will be promoted and accepted when integrated into population based cervical cancer screening programs.

## Figures and Tables

**Table 1:** Bivariate analysis of correlates of participants’ willingness to self-collect a vaginal sample for HPV-based cervix screening.

	Total	Not Willing ^1^	Willing ^2^	*p* Value
	4945	2371	2574	
Median Age (interquartile range (IQR))	53.8 (45.4, 61.5)	53.4 (45.0, 61.2)	54.1 (45.9, 61.6)	0.026
Education Level				
Incomplete post-secondary or less	1601 (32.4%)	854 (36.0%)	747 (29.0%)	<0.001
Complete college or higher	3308 (66.9%)	1496 (63.1%)	1812 (70.4%)	
Missing	36 (0.7%)	21 (0.9%)	15 (0.6%)	
Marital Status				
Living without a partner	1114 (22.5%)	524 (22.1%)	590 (22.9%)	0.762
Living with a partner	3792 (76.7%)	1829 (77.1%)	1963 (76.3%)	
Missing	39 (0.8%)	18 (0.8%)	21 (0.8%)	
Income				
Under or equal to $75,000	1498 (30.3%)	735 (31.0%)	763 (29.6%)	<0.001
Over $75,000	3009 (60.8%)	1380 (58.2%)	1629 (63.3%)	
Missing	438 (8.9%)	256 (10.8%)	182 (7.1%)	
I know more about HPV and cervical cancer now than I did before I participated in the study:				
Agree	1672 (33.8%)	766 (32.3%)	906 (35.2%)	0.071
Disagree	1458 (29.5%)	688 (29.0%)	770 (29.9%)	
Neutral	1703 (34.4%)	860 (36.3%)	843 (32.8%)	
Not Sure	98 (2.0%)	51 (2.2%)	47 (1.8%)	
Missing	14 (0.3%)	6 (0.3%)	8 (0.3%)	
Having an HPV test to screen for cervical cancer instead of a Pap smear is acceptable to me:				
Agree	3107 (62.8%)	1379 (58.2%)	1728 (67.1%)	<0.001
Disagree	573 (11.6%)	324 (13.7%)	249 (9.7%)	
Don’t Know	476 (9.6%)	238 (10.0%)	238 (9.2%)	
Neutral	775 (15.7%)	422 (17.8%)	353 (13.7%)	
Missing	14 (0.3%)	8 (0.3%)	6 (0.2%)	
Collecting my own sample for cervical cancer screening would be: Acceptable				
Acceptable	3356 (67.9%)	996 (42.0%)	2360 (91.7%)	<0.001
Unacceptable	1416 (28.6%)	1256 (53.0%)	160 (6.2%)	
Missing	173 (3.5%)	119 (5.0%)	54 (2.1%)	

^1^ Those who were neutral (12%), don’t know (4%), disagreed (17%), or strongly disagreed (15%) with the statement “I would be willing to collect my own sample/specimen for cervical cancer screening”. ^2^ Those who agreed (29%) or strongly agreed (23%) with the statement “I would be willing to collect my own sample/specimen for cervical cancer screening”.

**Table 2:** Multivariable analysis of correlates of participants’ willingness to self-collect a vaginal sample for HPV-based cervix screening.

	Adjusted Odds Ratio (OR)	95% Confidence Interval (CI)
Median Age	1.01 *	1.00, 1.02
Education Level		
Incomplete post-secondary or less	Reference	
Complete college or higher	1.31 **	1.12, 1.54
Marital Status		
Living without a partner	Reference	
Living with a partner	0.93	0.77, 1.12
Income		
Under or equal to $75,000	Reference	
Over $75,000	1.09	0.92, 1.29
I know more about HPV and cervical cancer now than I did before I participated in the study:		
Disagree	Reference	
Agree	0.98	0.82, 1.18
Neutral	0.83 *	0.69, 0.99
Not Sure	0.80	0.47, 1.37
Having an HPV test to screen for cervical cancer instead of a Pap smear is acceptable to me:		
Disagree	Reference	
Agree	1.45 *	1.15, 1.82
Don’t Know	1.30	0.95, 1.78
Neutral	0.96	0.73, 1.26
Collecting my own sample for cervical cancer screening would be: Acceptable		
Unacceptable	Reference	
Acceptable	17.9 **	14.9, 21.8

* *p* < 0.05, ** *p* < 0.001. This is based on a complete data set of 4327 (n of the model).

## Data Availability

The data presented in this study are available on request from the corresponding author. The data are not publicly available due to research ethics.

## Supplementary Materials

The following are available online at https://www.mdpi.com/article/10.3390/curroncol29060308/s1, File S1: Survey.
