# Supplementary material for: Willingness to Self-Collect a Sample for HPV-Based Cervical Cancer Screening in a Well-Screened Cohort: HPV FOCAL Survey Results
Source: Curr Oncol. 2022 May 26;29(6):3860–9. doi: 10.3390/curroncol29060308 (PMC9221715; doi:10.3390/curroncol29060308)
Supplement: Supplementary file 1 [file curroncol-29-00308-s001.zip › curroncol-1640108-supplementary.pdf]

Thank you for contributing to the HPV FOCAL Study! As you know the purpose of the study was to evaluate HPV testing for cervical cancer screening in BC. The results of the FOCAL study will be very important as Canadian programs consider adopting HPV testing to screen for changes that may lead to cervical cancer. We are interested to learn about your experience and understanding of HPV testing for cervical cancer screening. ***As a reminder, <u>ALL</u> women who completed a 4 year exit screen received both a Pap smear and an HPV test***

Your input is important to us and can help plan for the future of cervical cancer screening in BC. Please take a few moments of your time to complete the attached survey. You do not have to answer any questions you do not feel comfortable answering.

First we would like to ask about your HPV knowledge before and during the study.

1) *Please rate the following question according to how much you agree or disagree with the statement:* **I know more about HPV and cervical cancer now than I did before I participated in the study:**

☐ Strongly Disagree

☐ Disagree

☐ Neutral

☐ Agree

☐ Strongly agree

☐ Not Sure

2) **I was aware of my cervical screen (Pap and HPV test) results at completion of the study:**

☐ No

☐ Yes

☐ Not sure

3) *Please rate the following question according to how much you agree or disagree with the statement:* **I had enough time to ask my health care provider questions about HPV testing and/or my results.**

☐ Strongly Disagree

☐ Disagree

☐ Neutral

☐ Agree

☐ Strongly agree

☐ Don’t know

☐ Not applicable

4) *Please rate the following question according to how much you agree or disagree with the statement:*
I feel my health care provider was able to answer my questions about HPV:

☐ Strongly Disagree

☐ Disagree

☐ Neutral

☐ Agree

☐ Strongly agree

☐ Don’t know

☐ Not applicable

5) *Please rate the following statements regarding HPV testing:*
Having my health care provider collect a sample for HPV testing for cervical cancer screening would be:

	**<---**	**<--**	**<-**	**Neutral**	**->**	**-->**	**--->**	
Accurate	☐	☐	☐	☐	☐	☐	☐	Inaccurate
Safe	☐	☐	☐	☐	☐	☐	☐	Unsafe
Protect my health	☐	☐	☐	☐	☐	☐	☐	Harm my health
Acceptable	☐	☐	☐	☐	☐	☐	☐	Unacceptable

6) Which of the following have been important sources of information for you about HPV/HPV testing?

	**Not at all important**	**Not that important**	**Neutral**	**Somewhat important**	**Very important**
My health care provider	☐	☐	☐	☐	☐
BC Cancer Agency (including the BC Cancer Agency website)	☐	☐	☐	☐	☐
Government websites (ie: Canadian Cancer Society, Health Canada, Public Health Agency of Canada, BC Ministry of Health)	☐	☐	☐	☐	☐
Other websites (ie: WebMD, blogs, etc)	☐	☐	☐	☐	☐
Friends/family	☐	☐	☐	☐	☐
Social Media (ie: facebook, twitter, etc)	☐	☐	☐	☐	☐

Other comments:……

……… Next we would like to ask how you feel about HPV testing to screen for cervical cancer.

As a reminder, there are over 100 types of the human papillomavirus (HPV), of which about 40 affect the genital region. HPV is very common and most sexually active people will have an infection at some point in their lives, however it usually disappears without a person experiencing any symptoms. Only in cases where a cancer causing HPV type persists for many years, is there a risk that it may lead to cervical cancer.

7) *Please rate the following question according to how much you agree or disagree with the statement:* **Having an HPV test to screen for cervical cancer instead of a Pap smear is acceptable to me:**

☐ Strongly Disagree

☐ Disagree

☐ Neutral

☐ Agree

☐ Strongly agree

☐ Don't know

Comments:……..........................

8) **I would be willing to have an HPV test <u>every 4 or 5 years</u> instead of a Pap test every 3 years:**

☐ Strongly Disagree

☐ Disagree

☐ Neutral

☐ Agree

☐ Strongly agree

☐ Don't know

Comments: ……..........................

Evidence suggests that HPV testing is ideally started no earlier than 30 years of age. Should HPV testing be adopted in the province, it would be available for women starting at age 30 or 35. However, cervical cancer screening could still start at age 25 using the Pap smear as currently recommended in BC. **9) Receiving HPV testing for cervical cancer screening, starting at age 30 is acceptable to me.**

☐ Strongly Disagree

☐ Disagree

☐ Neutral

☐ Agree

☐ Strongly agree

☐ Don't know

10) If cervical cancer screening was to occur every 4 or 5 years, instead of every 3 years, I would be less likely to visit my health care provider for other health reasons.

☐ Strongly Disagree

☐ Disagree

☐ Neutral

☐ Agree

☐ Strongly agree

☐ Don’t know

Next we would like to understand some of your thoughts about HPV testing compared to the Pap test.

11) What would concern you most: 1) being told you had “abnormal Pap test results” or 2) being told you were “positive for HPV”? (*Check* <u>*ONE*</u> *only*)

☐ Neither would concern me

☐ Being told I have abnormal Pap test results

☐ Being told I have HPV

☐ Both would concern me equally

Please explain: ………

………

Next, we would like to learn about your experience of receiving HPV results.

12) If you tested HPV positive during the HPV FOCAL trial, or if you were to test positive for HPV, please rate your level of agreement with the following statements:

	**Strongly disagree**	**Disagree**	**Neutral/uncertain**	**Agree**	**Strongly Agree**
Although HPV is sexually acquired, having an infection that is sexually acquired does not concern me any differently than abnormal Pap results would.	☐	☐	☐	☐	☐

It would be important for me to know who gave me HPV.	☐	☐	☐	☐	☐
It would be important to me to determine when I got HPV.	☐	☐	☐	☐	☐
I think people might judge me for having HPV.	☐	☐	☐	☐	☐
I would feel comfortable telling my partner about my HPV positive result.	☐	☐	☐	☐	☐
I would not be concerned about transmitting HPV to a sexual partner.	☐	☐	☐	☐	☐
Receiving a positive HPV result would not affect my relationship with my sexual partner.	☐	☐	☐	☐	☐
Having HPV would not cause me any concern about developing cervical cancer.	☐	☐	☐	☐	☐
I would feel confident in the follow up and/or treatment my provider would recommend for my positive HPV result.	☐	☐	☐	☐	☐

Other comments:………. ………………
……..

13) *Please rate the following statements according to how much you agree or disagree:* **If I tested positive for HPV I felt/would feel:**

	**Strongly disagree**	**Disagree**	**Neutral/uncertain**	**Agree**	**Strongly agree**
Reassured	☐	☐	☐	☐	☐

Relieved	☐	☐	☐	☐	☐
Guilty	☐	☐	☐	☐	☐
Worried	☐	☐	☐	☐	☐
Upset	☐	☐	☐	☐	☐
Surprised	☐	☐	☐	☐	☐

Other comments: ……

14) If you were aware of your results at the end of the trial and they were negative/normal, how confident were you with the safety of these results?

☐ Very unconfident

☐ Somewhat unconfident

☐ Neutral

☐ Somewhat confident

☐ Very confident

☐ Didn't know my results

☐ Not applicable/results were positive

Next we would like to ask about your thoughts about the possibility of women collecting their own sample for HPV testing for cervical cancer screening by inserting a soft Q-tip or brush into the vagina that can then be returned to the laboratory and tested for the presence of HPV. With self-collection, a woman would not need to see a health care provider for pelvic exam for sample collection. Selfcollected specimens tested for HPV have been shown to be equivalent to HPV samples taken by a health care provider for detecting abnormalities of the cervix.

15) *Please rate the following statements regarding self-collected samples:* **Collecting my own sample for cervical cancer screening would be:**

	**<---**	**<--**	**<-**	**Neutral**	**->**	**-->**	**--->**	
Accurate	☐	☐	☐	☐	☐	☐	☐	Inaccurate

Safe	☐	☐	☐	☐	☐	☐	☐	Unsafe
Protect my health	☐	☐	☐	☐	☐	☐	☐	Harm my health
Acceptable	☐	☐	☐	☐	☐	☐	☐	Unacceptable

16) **I would be willing to collect my own sample/specimen for cervical cancer screening:**

☐ Strongly Disagree

☐ Disagree

☐ Neutral

☐ Agree

☐ Strongly agree

☐ Don't know

Comments: ……..
……….

Finally we'd like to ask some questions that may help us better understand associations between HPV and various other factors.

17) **What is the highest level of education you have achieved?**

☐ No formal education

☐ Some elementary school

☐ Completed elementary school

☐ Some high school

☐ Completed high school

☐ Some post-secondary training (Trades, college, university)

☐ Completed trade/vocational/apprenticeship program

☐ Completed college

☐ Completed university (bachelor degree)

☐ Completed university (master degree or higher)

18) **Please indicate your current marital status:**

☐ Single (living WITHOUT a partner)

☐ Single (living WITH a partner)

☐ Married or common law

☐ Divorced

☐ Separated (but still legally married)

☐ Widowed

19) **Not including new partners you have had since you completed the HPV FOCAL study, please indicate to the best of your recollection, how many male partners you have had vaginal intercourse with:**

☐ 0

☐ 1-10

☐ 11-49

☐ 50+

20) **Please enter the total years you have used the following hormonal birth control methods. If never used, please enter "0". (survey build, enter in months)**

**Method**	**Total years used**	**Please place a checkmark in this column if you used this method while participating in the HPV FOCAL study**
Oral contraceptive pill	_ _	☐
Contraceptive patch	_ _	☐
Contraceptive vaginal ring	_ _	☐
Hormonal IUD	_ _	☐
Injection (ie: Depoprovera)	_ _	☐

Morning after pill or emergency contraceptive pill	_ _	☐
Implant	_ _	☐

We are trying to better understand how use of specific substances (ie: alcohol, tobacco and marijuana) impacts a woman's potential to develop a long term HPV infection. These following questions help us determine if there is an association between HPV infection and different substances.

21) **Have you ever smoked cigarettes?**

☐ Yes (daily). For approximately how many years: _ _

☐ Yes (less than daily)

☐ Not at all

22) **Were you a regular cigarette smoker during your participation in the HPV FOCAL Study?**

☐ Every day…..Approximately how many cigarettes did you smoke per day? _ _

☐ Some days…Approximately how many days did you smoke each week? _ _

☐ Not at all

(Survey build). Only link to Q22 if answered "every day" or "some days" to Q21

23) **During your participation in the HPV FOCAL Study, how often did you use marijuana:**

☐ Never

☐ Less than once a month

☐ 1-3 times a month

☐ Once a week

☐ More than once a week

☐ Every day

Survey build: skip Q24 if answered "never" to Q23

24) **Please indicate the ways you used marijuana during the HPV FOCAL study:**

☐ Smoking

☐ Vaporizing

- ☐ Edible (ie: cakes, cookies, candy, drinks, etc)
- ☐ Pill/capsule
- ☐ Other

***25)* During your participation in the HPV FOCAL Study, how often did you drink alcoholic beverages?**

- ☐ Never
- ☐ Once a month or less
- ☐ 2-3 times a month
- ☐ Once a week
- ☐ 2-3 times a week
- ☐ 4-6 times a week
- ☐ Every day

***26)* Please estimate your total household income (everyone in your household, excluding roommates):**

- ☐ Less than $10,000
- ☐ $10,001-25,000
- ☐ $25,001-50,000
- ☐ $50,001-75,000
- ☐ $75,001-100,000
- ☐ More than $100,000

Thank you for completing this survey! Your responses are highly valued and impact guidelines and recommendations as the province of British Columbia considers changes to cervical cancer screening practices. If you have any questions or comments please feel free to contact the HPV FOCAL study at hpvfocal@bccancer.bc.ca

……
……

SURVEY BUILD: Link to a separate section where respondents can enter their name and contact info to be entered into a draw. We will offer an incentive for survey completion.
